# Comment on Bianco et al. Differences in Arrhythmia Detection Between Harvard Step Test and Maximal Exercise Testing in a Paediatric Sports Population. *J. Cardiovasc. Dev. Dis.* 2025, *12*, 22

**DOI:** 10.3390/jcdd12060225

**Published:** 2025-06-13

**Authors:** Daniel Neunhaeuserer, Francesca Battista, Giulia Quinto, Gino Degano, Andrea Gasperetti, Andrea Ermolao

**Affiliations:** 1 Sports and Exercise Medicine Division, Department of Medicine, University of Padova, Via Giustiniani 2, 35128 Padova, Italyandrea.ermolao@unipd.it (A.E.); 2 Sports and Exercise Medicine Division, University Hospital of Padova, Via Giustiniani 2, 35128 Padova, Italy

The article by Bianco M. et al. titled “Differences in Arrhythmia Detection Between Harvard Step Test and Maximal Exercise Testing in a Paediatric Sports Population” provides valuable insights into the efficacy of the Harvard Step Test (HST) and Maximal Exercise Testing (MET) in detecting arrhythmias in young athletes, also addressing the recovery phase [1]. However, some apparently conflicting outcomes when compared with a recent study addressing a similar research question should be more deeply discussed [2], also addressing possible study biases and limitations regarding the design of both studies, which may open a constructive discussion to enhance the robustness of these interesting findings, particularly for clinicians dealing with Italian pre-participation screening protocols.

While the outcomes of Quinto G. et al. should be interpreted considering that the comparison between HST and MET was carried out between independent unpaired samples, Bianco M. et al. transparently specified potential selection biases related to the specific inclusion criteria of the evaluated cohort [1,2]. Moreover, the study population includes athletes who were tested with an HST and MET as second-level evaluations for clinical reasons [1]. This methodological approach can influence the prevalence of arrythmias, which, thus, cannot be compared with general screening outcomes in young athletes. Furthermore, both the HST and MET were performed with the aim of achieving maximal effort, as determined by a Rating of Perceived Exertion (RPE) or >85% of the age-predicted maximum heart rate. In other words, HSTs have been specifically performed for reaching the highest intensities, thereby excluding submaximal HSTs, as frequently performed in real-world Italian pre-participation screening (i.e., 3 min at a rate of 30 steps/minute or until exhaustion, +2 min of recovery). This may theoretically potentiate possible selection bias, also limiting the generalisability of these results.

Furthermore, the inclusion of both treadmill and cycle-ergometer testing for the METs introduces additional variability in the study conducted by Bianco M. et al. [1]. Treadmill testing is known to lead to higher peak heart rates and may induce diverse arrhythmogenic stimuli compared to cycle-ergometer testing. Different test modalities could thus have further influenced the detection rates of arrhythmias during MET, and the authors may consider conducting specific additional sub-analyses. Standardising the type of ergometer used for METs could provide more consistent results. In addition, the statistical evaluation does not show reproducibility data for arrhythmias when the HST and MET are compared, which could be useful in order to investigate respective diagnostic power.

It is concluded that the HST detects arrhythmias more effectively than MET, particularly in males and during the recovery phase [1], which seems somehow in contrast with the study results found by our group, showing a lower occurrence of arrhythmias in evaluations performed with HST than with MET in young competitive athletes [2]. As Bianco M. et al. correctly discussed, far fewer individuals performing the HST reached maximal exercise intensities when compared to MET in the population investigated by Quinto G. et al. This is due to the fact that real-world data of Italian screening protocols are analysed in that study, where young athletes performing the HST do frequently not reach exhaustion, which is not required by the current screening protocols. Furthermore, the METs in the study by Quinto G. et al. are all performed on an inclined treadmill, leading to a similar type of activity during both testing protocols. Indeed, the main conclusion of the paper by Quinto G. et al. was related to the exercise intensity and not the test method. These differences in test characteristics and modalities should be considered and further investigated regarding arrhythmia detection, but the achievement of maximal exercise intensities seems crucial during stress testing [3].

In conclusion, while the articles by Bianco M. and Quinto G. et al. both contribute significantly to the field of sports cardiology and sports medicine [1], addressing the respective limitations, aforementioned biases and related perspectives with future research proposals would enhance the robustness and reliability of the studies’ findings. Given the crucial role of the Italian pre-participation screening model in the early detection of clinical conditions that could affect athletes’ health, we believe it is valuable to discuss the results of this and previous articles within a broader context [1,2,3]. This could support a potential revision, amendments and implementation adaptations for national screening protocols for athletes, considering the advancements in sports and exercise medicine over the past 40 years [4].

## Abbreviations

The following abbreviations are used in this manuscript:

HST	Harvard Step Test
MET	Maximal Exercise Testing
